# Lysosomal diseases: Overview on current diagnosis and treatment

**DOI:** 10.1590/1678-4685-GMB-2018-0159

**Published:** 2019-04-25

**Authors:** Fabiano de Oliveira Poswar, Filippo Vairo, Maira Burin, Kristiane Michelin-Tirelli, Ana Carolina Brusius-Facchin, Francyne Kubaski, Carolina Fischinger Moura de Souza, Guilherme Baldo, Roberto Giugliani

**Affiliations:** 1 Medical Genetics Service, Hospital de Clínicas de Porto Alegre, Porto Alegre, RS, Brazil; 2 Department of Genetics, Universidade Federal do Rio Grande do Sul, Porto Alegre, RS, Brazil; 3 Postgraduate Program in Genetics and Molecular Biology, Universidade Federal do Rio Grande do Sul, Porto Alegre, RS, Brazil; 4 Center for Individualized Medicine, Mayo Clinic, Rochester, MN, USA; 5 Department of Clinical Genomics, Mayo Clinic, Rochester, MN, USA; 6 Postgraduate Program in Physiology, Universidade Federal do Rio Grande do Sul, Porto Alegre, RS, Brazil; 7 Department of Physiology and Pharmacology, Universidade Federal do Rio Grande do Sul, Porto Alegre, RS, Brazil

**Keywords:** Lysosomal storage diseases, neonatal screening, hematopoietic stem cell transplantation, enzyme replacement therapy, gene therapy

## Abstract

Lysosomal diseases (LDs), also known as lysosomal storage diseases (LSDs), are a heterogeneous group of conditions caused by defects in lysosomal function. LDs may result from deficiency of lysosomal hydrolases, membrane-associated transporters or other non-enzymatic proteins. Interest in the LD field is growing each year, as more conditions are, or will soon be treatable. In this article, we review the diagnosis of LDs, from clinical suspicion and screening tests to the identification of enzyme or protein deficiencies and molecular genetic diagnosis. We also cover the treatment approaches that are currently available or in development, including hematopoietic stem cell transplantation, enzyme replacement therapy, small molecules, and gene therapy.

## Introduction

Lysosomes are membrane-bound organelles, which contain, among other components, hydrolytic enzymes that operate in an acidic environment (Sabatini and Adesnik, 2014). Lysosomes are capable of digesting all types of macromolecules and participate in the breakdown of both extracellular and intracellular components that are targeted to them through the processes of endocytosis or autophagy, respectively.

Lysosomal diseases, also known as lysosomal storage diseases, are a heterogeneous group of diseases caused by defects in lysosomal function (Valle *et al.*, 2014). Most LDs result from a deficiency in lysosomal hydrolases (*e.g.*, alpha-galactosidase in Fabry disease). Alternatively, LDs may be caused by deficiencies in lysosomal membrane-associated transporters (*e.g.*, cystinosin in cystinosis) or other non-enzymatic proteins (*e.g.*, CLN3 in Batten disease). According to the WORLDSymposia® official list of lysosomal diseases, 66 clinical conditions related to 53 distinct genes are recognized as LDs (WORLDSymposium, 2018).

Although individually very rare, the incidence of LDs as a group is estimated to be as high as 1 in 4000 in some countries (Giugliani *et al.*, 2017a). The exact prevalence is difficult to estimate, considering the clinical heterogeneity of LDs, which may lead to missed diagnoses. According to Medical Genetics Service of the Hospital de Clínicas de Porto Alegre data, the investigation of high-risk subjects led to 3,512 LD diagnoses in Brazil from 1982 to 2017 (Table 1).

**Table 1:** Lysosomal storage diseases diagnosed from 1982 to 2017 by the Reference Laboratory of Inborn Errors of Metabolism, Medical Genetics Service, Hospital de Clinicas de Porto Alegre, Brazil.

Lysosomal storage disease	Number of confirmed diagnoses
Mucopolysaccharidoses	
Mucopolysaccharidosis type I	**262**
Mucopolysaccharidosis type II	**413**
Mucopolysaccharidosis type IIIA	**67**
Mucopolysaccharidosis type IIIB	**104**
Mucopolysaccharidosis type IIIC	**68**
Mucopolysaccharidosis type IVA	**193**
Mucopolysaccharidosis type IVB	**13**
Mucopolysaccharidosis type VI	**281**
Mucopolysaccharidosis type VII	**22**
Multiple sulfatase deficiency	**9**
Glycoproteinoses	
Aspartylglucosaminuria	**1**
Fucosidosis	**4**
Galactosialidosis	**19**
α-Mannosidosis	**9**
Mucolipidosis II/III	**41**
Sialidosis	**14**
Sphingolipidoses	
Fabry disease	**109**
Gaucher disease	**756**
GM1 gangliosidosis	**181**
GM2 Tay-Sachs disease (44% B1)	**144**
GM2 Sandhoff disease	**30**
Krabbe disease	**109**
Metachromatic leukodystrophy	**164**
Niemann-Pick type A/B disease	**225**
Other LDs	
Lysosomal acid lipase deficiency	**11**
Neuronal ceroid lipofuscinosis 1 (CLN1)	**6**
Neuronal ceroid lipofuscinosis 2 (CLN2)	**43**
Niemann-Pick type C	**161**
Pompe disease	**52**
Salla disease	**1**
TOTAL	**3512**

* Classified as proposed by Kingma *et al.*, 2015.

Interest in the LD field is growing as more conditions are now treatable or are expected to be treatable in the near future by distinct approaches including hematopoietic stem cell transplantation, enzyme replacement, small molecules, and gene therapy (Beck, 2018). Research in this field is also important, as molecular pathways related to lysosomal disease pathophysiology are increasingly recognized as being impaired in more common conditions including Parkinson’s disease and aging (Lloyd-Evans and Haslett, 2016).

## Clinical suspicion

LD symptomatology depends on the stored substrate and organs affected by this accumulation. Usually, substrate accumulation occurs in the organs where they are synthesized (*e.g.*, liver, spleen, bone, etc.), which partially explains the involvement of different organs. Issues with the targeting of enzymes to lysosomes, defective membrane proteins, and abnormal excretion of substrates may also cause lysosome enlargement and functional impairment. Thus, the wide range of symptoms in LD may be explained by the activation of several deleterious processes, such as the release of acid hydrolases into the cytoplasm causing cellular damage, the dysregulation of apoptosis or the abnormal accumulation of lipids causing defective transport of substrates into and out of the lysosomes.

LDs are traditionally classified according to the substance that accumulates abnormally. However, this classification is merely for convenience, since there is overlap in the substrate specificities of enzymes. The major categories of LDs are mucopolysaccharidoses, mucolipidoses, sphingolipidoses, oligosaccharidosis, and neuronal ceroid lipofuscinoses (Giugliani *et al.*, 2017b).

There are some phenotypic features that should raise the suspicion of LD. For example, if a patient presents with coarse facial features, hepatosplenomegaly, and skeletal abnormalities, one should suspect mucopolysaccharidosis, mucolipidosis, or oligosaccharidosis, remembering that there are subtypes associated with neurological impairment or corneal clouding, which could lead to a more precise diagnosis. A very specific sign, such as a “cherry red” spot in the retina, indicates that the physician should prioritize GM1- and GM2-gangliosidosis as a possible differential diagnosis. Angiokeratomas are almost specific for Fabry disease and fucosidosis, for instance. A patient with anemia, thrombocytopenia, and hepatosplenomegaly should be evaluated for Gaucher and Niemann-Pick type B diseases. For any patient presenting with neurodegeneration and vision issues at any age, the clinical team should suspect an underlying neuronal ceroid lipofuscinosis. For three out of the 14 neuronal ceroid lipofuscinosis types there is an enzymatic test clinically available. For the other types, genetic analysis or electron microscopy of lymphocytes or fibroblasts is advised.

Examples of diseases within each of the categories and the major signs and symptoms seen in patients with diseases in each group are summarized in Table 2.

**Table 2:** Major signs and symptoms of LDs.

Major LD category	Examples	Major signs and symptoms^*^
Mucopolysaccharidoses	MPS I (IH, IS, and IH/S); MPS II;, MPS III (A, B, C, and D); MPS IV (A and B); MPS VI; MPS VII, MPS IX	Coarse facial features, hepatosplenomegaly, corneal clouding, skeletal abnormalities, joint limitation, and short stature; progressive mental retardation occurs in some types
Mucolipidoses	Type I; Type II; Type III; Type IV	Coarse facial features, hepatosplenomegaly, dysostosis multiplex, finger contractures, scoliosis, short stature; progressive mental retardation occurs in some types
Sphingolipidoses	GM2-gangliosidoses; Niemann-Pick (types A, B, and C); Gaucher disease (types I, II, and III); Fabry disease; Metachromatic leukodystrophy; Krabbe disease; Farber lipogranulomatosis	Neurodegeneration, “cherry red” spot in the retina, hepatosplenomegaly, pulmonary involvement, gaze palsy, ataxia, bone changes, paresthesias, angiokeratomas, renal failure
Oligosaccharidoses	α-mannosidosis; β-mannosidosis; fucosidosis; aspartylglucosaminuria; Schindler disease; ISSD; Salla disease; Galactosialidosis; GM1-gangliosidosis	Coarse facial features, dysostosis multiplex; “cherry red” spot in the retina, hepatosplenomegaly, mental retardation, ataxia, hearing loss, angiokeratoma
Neuronal ceroid lipofuscinoses	Types 1 to 14	Neurodegeneration, vision issues, seizures, ataxia

IH: Hurler; IS: Sheie; IH/S: Hurler-Scheie; ISSD: Infantile sialic acid storage disease.

* May not be present in all diseases in the same category.

## Considerations regarding the diagnosis of LDs

### Biomarkers and screening tests

In the LDs associated with enzyme deficiencies, diagnosis is usually performed by the direct measurement of the activity of the enzyme associated with the disease. To identify which enzyme assay should be performed, it is useful to measure biomarkers, indicated by the clinical picture. Biomarkers may be especially important when the LD is caused by the deficiency of a non-enzymatic protein, which could be difficult to measure.

The measurement of biomarkers in different biological samples (blood, urine, cerebrospinal fluid) can be carried out either before or simultaneously with the enzyme activity measurement. A biomarker is generally an analyte that indicates the presence and/or extent of a biological process, which is in itself usually directly linked to the clinical manifestations and outcome of a particular disease (Bobillo Lobato *et al.*, 2016). To assess the effectiveness of therapies, it is helpful to use biomarkers that allow us to analyze the evolution of the disease over time, determining how the accumulation of products diminishes. Biomarkers are a key component not only of the diagnosis, but also for monitoring patients and for choosing the best therapeutic option in each case. Biomarkers are also important in the case of pseudo-deficiencies, as they can provide information about the functional consequences of the detected enzyme abnormality.

Biomarkers may be analyzed qualitatively or quantitatively. Qualitative analyses (thin-layer chromatography, electrophoresis, spot tests, etc.), allow the identification of biomarkers but with low sensitivity and specificity. For instance, thin-layer chromatography of oligosaccharides is generally used but should soon be replaced by quantitative methods (Raymond and Rinaldo, 2013). For quantitative analyses, it is possible to use colorimetric methods, but tandem mass spectrometric methods seem to be most promising (Blau *et al.*, 2008).

For Fabry disease (FD), the analysis of globotriaosylsphingosine (lyso-Gb3) is preferable to globotriaosylceramide (Gb-3), because there is not a clear correlation between Gb3 levels and the clinical manifestation or severity of the disease. DBS provides a convenient, sensitive, and reproducible source to measure lyso-Gb3 levels for diagnosis, initial phenotypic assignment, and therapeutic monitoring in patients with FD (Nowak *et al.*, 2017a). Furthermore, it has been proven that lyso-Gb3 in plasma is a useful biomarker for the diagnosis and treatment of FD heterozygotes (Nowak *et al.*, 2017b). Proteinuria and creatinine are practical biomarkers of renal damage. Troponin I and high-sensitivity assays for cardiac troponin T can identify patients with cardiac lesions, but new cardiac imaging techniques are necessary to detect incipient damage (Beirão *et al.*, 2017).

The classical biomarker for Gaucher disease (GD) is chitotriosidase (ChT). ChT activity has been shown to correlate well with various clinical parameters and has been used to monitor and adjust the treatment, despite being not specific for GD. It should be mentioned that there are a significant number of individuals in the general population (~1:20) with low chitotriosidase activity due to a common polymorphism. One alternative option for these cases is the pulmonary and activation-regulated chemokine (CCL18/PARC) (Bobillo Lobato *et al.*, 2016). Another biomarker for GD is glucosylsphingosine, (proposed by Rolfs *et al.*, 2013), which is considered more specific than chitotriosidase or CCL18. Measured with LC-MS/MS, glucosylsphingosine achieved 100% specificity in identifying Gaucher patients (Rolfs *et al.*, 2013). The plasma biomarkers macrophage inflammatory protein 1-alpha and 1-beta (MIP-1α and MIP-1β) (van Breemen *et al.*, 2007), and cathepsin K have been used to study bone disease. Another alternative to chitotriosidase is osteopontin, which seems to have great potential as a biomarker for GD, although further investigation is still necessary (Vairo *et al.*, 2015).

Psychosine (PSY, galactosylsphingosine) has been suggested as a biomarker for the presence and progression of Krabbe disease (KD). PSY can be analyzed in blood, DBS, and cerebrospinal fluid. The psychosine concentration in patients with the infantile form of KD is at least four-fold higher than in asymptomatic newborns with low galactosylcerebrosidase activity, and nearly one order of magnitude greater than in healthy newborns (Bobillo Lobato *et al.*, 2016). PSY measurement in DBS could serve as a second tier assay in newborn screening for KD, simplifying and reducing the cost of follow-up protocols (Turgeon *et al.*, 2010). Quantitative analysis of diffusion tensor imaging (DTI) scalars, especially radial diffusivity and fractional anisotropy, has been shown to be a sensitive in vivo biomarker of white matter microstructural damage in KD (Poretti *et al.*, 2016).

For mucopolysaccharidoses (MPS), the investigation can start with urinary screening tests. Glycosaminoglycans (GAGs) are the most common and widely used biomarkers for MPS and several qualitative and quantitative methods have been used to-date (alcian blue, toluidine blue, paper and thin layer chromatography, gas chromatography, high-pressure liquid chromatography, capillary electrophoresis, 1,9-dimethylmethylene blue, carbazol, enzyme-linked immunosorbent assay, mass spectrometry, and others). Sensitivity and specificity of the dye-spectrometric and TLC methods are not sufficient to detect all types of MPS, especially MPS III and MPS IV (Kubaski *et al.*, 2017). Many studies have reported the analysis of glycosaminoglycan fragments by tandem mass spectrometry as a potential biomarker for MPS. Recently, a new quantitative UPLC–MS/MS method for heparin sulfate (HS), dermatan sulfate (DS), and chondroitin sulfate (CS) has taken advantage of equipment that is available at some clinical laboratories with basic triple quadrupole MS/MS systems. This method allows the determination of urinary levels of these biomarkers and facilitates diagnosis for patients with MPS I, II, III, IVA, and VI, as well as other lysosomal storage disorders. Langereis *et al.* (2015) adapted GAG quantification protocols by adding KS to provide a multiplex assay not only for the diagnosis of MPS but also for Mucolipidoses II and III.

Other MPS biomarkers in urine were identified using proteomics: β-galactosidase, collagen type I, fatty acid-binding protein 5, nidogen-1, cartilage oligomeric matrix protein, insulin-like growth factor binding protein 7, and Heg1. These compounds demonstrate a relationship between biomarker concentrations and disease severity (Bobillo Lobato *et al.*, 2016).

Sphingomyelin is elevated in Niemann-Pick diseases [both acid sphingomyelinase deficiency (ASMD, NP-A, NP-B) and Niemann-Pick C (NP-C)], but is not a reliable biomarker due to the overlap between the levels observed in patients and healthy controls. Lysosphingomyelin (lyso-SPM) levels in DBS seem to be a good alternative to sphyngomyelin. However, in patients with ASMD deficiency, lyso-SPM concentration does not correlate with the amount of residual enzyme activity in DBS or with patient age. The analysis of chitotriosidase or filipin staining of free cholesterol in fibroblasts lacks sensitivity and specificity for NP-C detection (Bobillo Lobato *et al.*, 2016). Recently, two metabolites that are markedly increased in NP-C patients have been identified as biomarkers. Higher levels of cholestane-3β, 5α, 6β-triol (C-triol) and 7-ketocholesterol (7-KC) are present in the plasma of NP-C patients when compared to plasma from patients with other LDs or control subjects. The concentration of these biomarkers correlates directly with the disease state, and they are specific to NP-C (Hammerschmidt *et al.*, 2018). Additional biomarkers have been described including 24(*S*)-hydroxycholesterol, which is reduced in the plasma and cerebrospinal fluid (CSF) (Tortelli *et al.*, 2014), bile acids in plasma, DBS, and urine; calbindin D, a compound found in cerebrospinal fluid (Bradbury *et al.*, 2016), and lyso-sphingomyelin-509 in plasma (Giese *et al.*, 2015).

Tetrasaccharide glucose (Glc4) is the most well-known biomarker for Pompe disease (PD) but is not specific, and its use for diagnostic purposes may be limited (Young *et al.*, 2009). The most frequently used technique is high-performance liquid chromatography with ultraviolet detection (HPLC/UV) due to its efficiency and availability in laboratories. There is a good correlation between urinary excretion of Glc4 and response to therapy (Manwaring *et al.*, 2012). Two other serum biomarkers, myostatin and insulin-like growth factor I (IGF-I), can be used for Pompe diesase, and increase after treatment (Bobillo Lobato *et al.*, 2016).

### Identification of the enzyme or protein deficiencies

**Figure 1:**
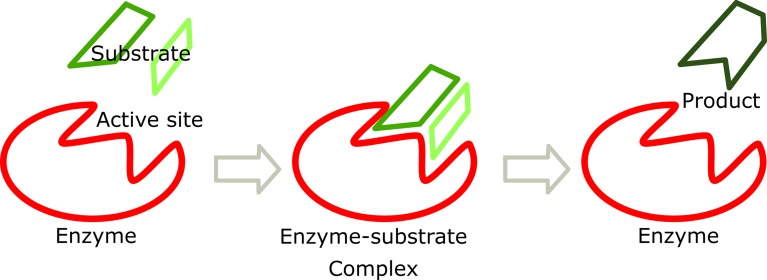
Hypothetical scheme of an enzymatic reaction. Point mutations in the gene encoding an enzyme may alter its enzymatic activity leading to substrate accumulation and a lack of product. In addition, it may also cause the accumulated substrate to follow an alternative route. This is the cause of many LDs.

Enzymes are proteins that catalyze chemical reactions and have high specificity for their substrates. This specificity allows the enzymes to be used to quantify their substrates. Additionally, the substrates can be used to determine the amount of enzyme present in a biological sample. Substrate binding occurs at the “active site” (Figure 1). The reaction rate will become maximal when the active sites of the enzyme molecules are occupied. Important variables to determine the enzymatic activity include temperature, pH, substrate concentration, cofactors, and the use of direct or indirect reactions to quantify the enzyme (Nelson *et al.*, 2014).

There are two important ways to determine the amount of enzyme in biological fluids. Most commonly, enzymes are quantified by determining their enzymatic activity by measuring the rate of a reaction catalyzed by the enzyme. In enzyme activity assays, some methods use endpoint quantification, determining the concentration of the substrate or product at a specific time after the addition of the sample. The biological fluids used for the enzymatic investigation of LDs include plasma, serum, leukocytes, cultured fibroblasts, dried blood spots (DBS), chorionic villi, amniotic fluid, and cultured amniocytes, among others (Henry and Gubert, 2008).

Most enzymatic assays for LD research rely on spectrofluorometry, which uses enzyme-specific substrates with a fluorogenic radical to generate a fluorophore product that will absorb energy at a specific wavelength and then emit it at another longer wavelength to determine the quantity of product produced. Spectrophotometry is also a widely used technique based on chromophores that excite themselves and emit colors depending on the energy released by the change from the basal to the excited state (Burns, 2016).

### Molecular genetic diagnosis

Precise molecular diagnosis (MD) is of great importance for LDs, not only to confirm the enzymatic diagnosis but also to ascertain a definitive diagnosis in complex situations.

Classification of disease severity based on the molecular defects may be useful when the enzyme deficiency and clinical information do not allow for a clear distinction between severe and more attenuated forms, and in cases where the specific enzyme shows high residual activity in affected patients and low enzymatic activity in unaffected patients (pseudodeficiencies). Moreover, MD is useful for those showing multiple enzyme deficiencies (*e.g.*, multiple sulfatase deficiency and mucolipidoses II and III,), and for confirmation of the diagnosis in X-linked conditions where the specific enzyme deficiency is not informative (females with FD), due to the overlap of the enzyme activities with the normal controls range. In addition, MD is essential for confirmation of the diagnosis in cases where the functional defect does not involve an enzyme deficiency (*e.g.*, neuronal ceroid lipofuscinosis and Niemann-Pick C disease) (Filocamo and Morrone, 2011).

MD can be made using DNA or RNA and utilizes a range of different molecular approaches, such as Sanger sequencing, restriction fragment length polymorphism (RFLP) analysis, amplification-refractory mutation system (ARMS), multiplex ligation-dependent probe amplification (MLPA), real-time PCR, and high-resolution melting. Currently, massive parallel sequencing technology, also known as next-generation sequencing (NGS), allows for the sequencing of large genomic regions in a short time period at relatively low cost, replacing the traditional analysis of individual genes and exon-by-exon sequencing. NGS applications include the sequencing of PCR-amplified genomic regions, whole-exome sequencing (WES), and whole-genome sequencing (WGS).

DNA sequencing is the primary clinical technique to identify mutations in LDs, but sequencing often does not detect intragenic or whole-gene deletions/duplications. Therefore, comparative genomic hybridization (CGH) using oligonucleotide arrays has been implemented in cytogenetic and molecular diagnostic laboratories as a robust, rapid, and sensitive assay for detecting targeted gene deletions (Brusius-Facchin *et al.*, 2014).

Since there are many lysosomal disorders that do not result from lysosomal enzyme deficiencies detected by clinically available tests, molecular testing must be considered as an important tool for the diagnosis of LDs. In this sense, WES has been reported as an important approach to diagnose LDs with unspecific phenotypes (Vairo *et al.*, 2017). Moreover, the determination of the genotype can be helpful in prenatal diagnosis, carrier detection, and for therapy options choice.

### Neonatal screening

Many studies suggest that it is feasible to screen for up to 10 LDs by measuring lysosomal enzymatic activities in DBS. DNA sequencing is not currently a first-tier option for newborn screening (NBS), although it has been considered as a complementary approach in some cases. The use of biomarkers is also not moving forward as a first-tier option for NBS of LDs, either because the analysis time per sample is too long for high-throughput NBS, or due to the high false-positive rates (3–5% in some reports). However, these methods are expected to be extremely valuable for second-tier analyses (in positive cases identified by enzyme activity assay), especially when the same DBS can be used, which would avoid patient recall and parental anxiety. As an example, for metachromatic leukodystrophy, the most promising approach for NBS is the analysis of sulfatides in DBS by MS/MS, since arylsulfatase A cannot be measured in DBS and its pseudodeficiency is quite common (Schielen *et al.*, 2017). As pilot studies have been completed worldwide and knowledge on the prevalence of these diseases increases, several national (or regional) screening programs have been adding LDs to their testing portfolio. In Taiwan, Pompe, Fabry, Gaucher, MPS-I, MPS-II, MPS-IVA, and MPS-VI are included in the national screening program. Some states in US, such as New York, Ohio, and Kentucky have included LDs to the NBS programs, and others are about to start screening for them. In Europe, only a few countries have started screening for LDs (Schielen *et al.*, 2017). A major concern about screening for these disorders is the presence of pseudodeficiencies that can cause a burden to the families and health systems. Another important matter is how to follow-up and/or treat the patients predicted to have late-onset forms. All these issues should be taken into account when discussing the screening for LDs.

## Considerations about treatment

### Hematopoietic stem cell transplantation

Hematopoietic stem cell transplantation (HSCT) is a known treatment for LDs due to the remarkable properties of self-renewing enzyme-producing cells to secrete the deficient enzyme and colonize enzyme-deficient tissues, allowing constant intercellular enzyme exchange. In this cross-correction process, secreted enzymes can be taken up from the reticuloendothelial system by deficient cells via the mannose-6-phosphate (M6P) or mannose receptors and transported to lysosomes where substrates can be properly degraded (Neufeld and Fratantoni, 1970; Kornfeld, 1992; Lund, 2013; Macauley, 2016; Mikulka and Sands, 2016; Jiang *et al.*, 2017). The main sources for HSCT are bone marrow (BM), peripheral stem cells (PSC), and cord blood (CB) (Aldenhoven *et al.*, 2015; Jiang *et al.*, 2017).

HSCT is considered a standard of care treatment for MPS type I, and it has been suggested for the treatment of metachromatic leukodystrophy (Boucher *et al.*, 2015; Boelens and van Hasselt 2016; Lum *et al.*, 2017; Parini *et al.*, 2017). Several reports have also indicated variable benefits of HSCT for MPS II (Barth *et al.*, 2017; Kubaski *et al.*, 2017), MPS VI (Behfar *et al.*, 2017), MPS IVA (Chinen *et al.*, 2014; Yabe *et al.*, 2016), MPS VII (Yamada *et al.*, 1998; Montaño *et al.*, 2016), Krabbe disease (Langan *et al.*, 2016; Maher and Yeager, 2016; Mikulka and Sands, 2016; Wright *et al.*, 2017), and fucosidosis (Jiang *et al.*, 2017).

**Figure 2:**
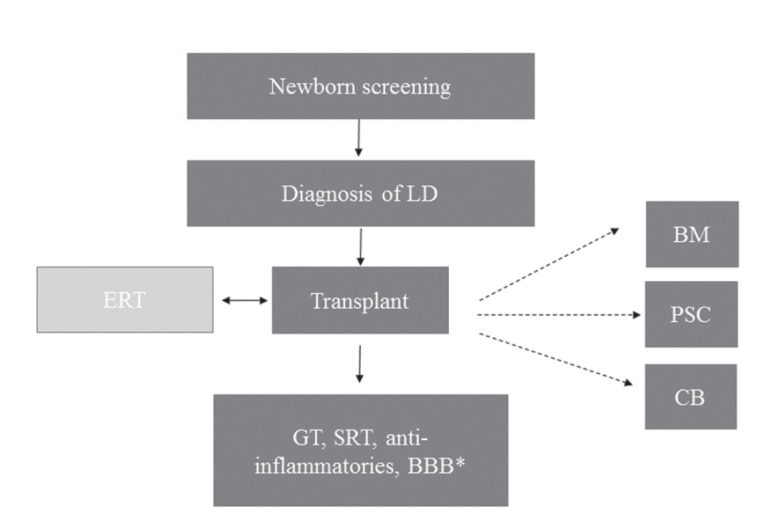
Tentative workflow for HSCT combined with newborn screening. After diagnosis of LDs, patients can receive a transplant as early as two weeks of age. ERT may start prior to the transplant and can be continued for the first few months until full chimerism is achieved. HSCT can also be combined with gene therapy, substrate reduction therapy, anti-inflammatories, and molecules that increase blood-brain barrier permeability to improve clinical outcomes. LD: lysosomal disorder; ERT: enzyme replacement therapy; BM: bone marrow; PSC: peripheral stem cell; CB: cord blood; GT: gene therapy; SRT: substrate reduction therapy; BBB: blood brain barrier; BBB*: molecules that increase BBB permeability.

HSCT is not a curative treatment for most LDs. However, it can slow disease progression and improve survival rates and quality of life for several of these disorders. The most crucial factor for improved outcomes is for the transplant to be performed as early as possible, ideally while patients are still asymptomatic. Newborn screening will greatly improve early detection of patients, allowing early transplantation that could be performed ideally before two weeks of age (Wright *et al.*, 2017) (Figure 2). For the first time, Barth *et al.* (2017) have recently demonstrated good HSCT outcomes in an MPS II patient transplanted at 70 days of age.

An advantage of HSCT over enzyme replacement therapy (ERT) is the fact that donor cells can cross the blood-brain barrier (BBB) and thus improve neurological impairment. However, CNS repopulation is a very slow process, which usually takes several months to occur, thus justifying the need for early HSCT to improve CNS impairment (Maher and Yeager, 2016). Finally, it should be mentioned that HSCT could potentially be combined with ERT, gene therapy (GT), substrate reduction therapy, and molecules that increase BBB permeability in order to improve clinical outcomes (Mikulka and Sands, 2016; Macauley, 2016).

### Enzyme replacement therapy

Lysosomes are cytoplasmic organelles that contain a variety of hydrolases. A genetic deficiency in the enzymatic activity of one of these hydrolases will lead to the accumulation of the material meant for lysosomal degradation (Ferreira and Gahl, 2017).

ERT was first successfully administered to humans with LDs over 25 years ago and was the first therapy that directly addressed the underlying mechanism causing a genetic disease. ERT is based on the intravenous infusion of a recombinant enzyme (similar to the natural one), which is taken up into the cell through membrane receptors (typically mannose-6-phosphate receptors) and replaces the catalytic action of the missing or non-functional lysosomal enzyme (Ortolano *et al.*, 2014). A functional version of the missing or hypoactive enzyme is produced by genetically engineered cell lines in a cGMP-compliant fashion. The purified enzyme may sometimes be modified to better target the lysosomal targeting pathways (Rastall and Amalfitano, 2017).

GD was the first LD for which the recombinant human β-glucocerebrosidase enzyme was developed and approved by the FDA in 1991 (Barton *et al.*, 1991). Development of ERT for FD followed (Biegstraaten *et al.*, 2015). ERT has also been developed for PD, MPS types I, II, IVA, VI, and VII, and lysosomal acid lipase deficiency, becoming the mainstay of treatment for individuals affected by these disorders (Rastall and Amalfitano, 2017). ERT has been the most successful treatment for LDs to-date and is currently being explored for other conditions such as acid sphingomyelinase deficiency (Wasserstein *et al.*, 2018) and alpha-mannosidosis (Borgwardt *et al.*, 2013). Table 3 shows a list of LDs and different forms of ERT.

**Table 3:** Approved enzyme replacement therapies.

Disease	Generic name	Brand name	Dose	Delivery
Gaucher type I	Imiglucerase	Cerezyme®	60 Units/kg (every other week)	I.V. infusion
	Taliglucerase alfa	Elelyso®	60 Units/kg (every other week)	
	Velaglucerase alfa	Vpriv®	60 Units/kg (every other week)	
Fabry disease	Agalsidase beta	Fabrazyme®	1 mg/kg (every other week)	I.V. infusion
	Agalsidase alfa	Replagal®	0.2 mg/kg (every other week)	
Pompe disease	Alglucosidase alfa	Myozyme®	20 mg/kg (every other week)	I.V. infusion
	Alglucosidase alfa	Lumizyme®		
MPS I - Hurler, Hurler-Scheie and Scheie	Laronidase	Aldurazyme®	0.58 mg/kg (once per week)	I.V. infusion
MPS II – Hunter Syndrome	Idursulfase	Elaprase®	0.5 mg/kg (once per week)	I.V. infusion
MPS VI - Maroteaux-lamy syndrome	Galsulfase	Naglazyme®	1 mg/kg (once per week)	I.V. infusion
MPS IVA – Morquio A syndrome	Elosulfase alfa	Vimizim®	2 mg/kg (once per week)	I.V. infusion
Lysosomal acid lipase deficiency	Sebelipase alfa	Kanuma®	1 mg/kg (every other week)	I.V. infusion
Late infantile neuronal ceroid lipofuscinosis type 2 (CLN2)	Cerliponase alfa	Brineura®	300 mg (every other week)	Intraventricular
MPS VII – Sly syndrome	Vestronidase alfa	Mepsevii®	4 mg/kg (every other week)	I.V. infusion

ERT represents a major advancement in the treatment of genetic disorders. However, the development and implementation of large-scale ERTs has unmasked several challenges in the treatment of LDs (Rastall and Amalfitano, 2017). Recombinant enzymes are very expensive and not all patients may benefit from them. Due to intravenous ERT not being efficacious in controlling CNS disease manifestations, the BBB limitation has been addressed with different routes of administration, including intracerebroventricular (ICV) and intrathecal (IT) delivery. The ICV approach has been approved for neuronal ceroid lipofuscinosis II (CLN2), and trials are ongoing to prove its safety and efficacy in several other conditions (Ortolano *et al.*, 2014). IT ERT was first used in a MPS patient in Brazil (Munoz-Rojas *et al.*, 2008) and is currently in clinical trials for MPS I (IT), MPS II (IT and ICV), and MPS IIIB (ICV) (Dickson *et al.*, 2015; Muenzer *et al.*, 2016; Muschol *et al.*, 2018).

A promising strategy to enable enzymes to penetrate the blood-brain barrier is the development of fusion proteins, in which enzyme molecules are attached to peptides or peptidomimetic antibodies that can cross this barrier through receptor-mediated endocytosis and act as so-called molecular “Trojan horses.” One such approach includes the use of a human insulin receptor monoclonal antibody, which has been tested in rhesus monkeys and is able to deliver sufficient amounts of α-iduronidase, iduronate-2-sulphatase, sulphamidase, and α-*N*-acetylglucosaminidase to the CNS (Pardridge *et al.*, 2018).

It is important to note that ERT requires lifelong, repeated infusions of large quantities of the respective exogenous enzyme. The amounts of enzyme that must be infused to effectively treat all affected cells, tissues, or organs in an LD patient can be quite large, and producing this much enzyme using current-GMP-compliant production methods can be very expensive and is likely limiting. Furthermore, ERT relies on active transport to eventually enter the cell and then the lysosome. These are likely rate-limiting steps; thus, despite massive infusions of recombinant enzyme, only a small proportion may actually make it into the lysosome (Jurecka and Tylki-Szymanska, 2015). Finally, due to individual genetic backgrounds, ERT can potentially elicit an immune response against the recombinant enzyme itself with higher titers correlating with poorer responses to the therapy (van Gelder *et al.*, 2015).

### Small molecule therapy

Small molecules are a more-recent development in the field of specific treatments for LDs. Instead of replacing a deficient enzyme, as in ERT, small molecules address the underlying mechanisms of the LDs by different methods including the reduction of the amount of substrate and the stabilization of the endogenous enzyme as a pharmacological chaperone. These therapies may have important advantages, including the possibility of being administered orally, the ability to cross the blood-brain barrier, a lack of hypersensitivity reactions, and lower manufacturing costs. Table 4 summarizes the small molecules currently approved for the treatment of LDs.

**Table 4:** Approved small molecule-based therapies for lysosomal diseases.

Disease	Compound	Class	Safety	Efficacy evidence
Gaucher disease	Miglustat	Substrate reduction therapy	Osmotic diarrhea and weight loss observed in the majority of patients. Peripheral neuropathy and tremor may occur	Reduction of glycosphingolipids, improvement in anemia and thrombocytopenia. Less effective, in general when compared to ERT and eliglustat
	Eliglustat	Substrate reduction therapy	Headache, arthralgia, nasopharyngitis, upper respiratory infection, diarrhea and dizziness were reported. Caution recommended in patients with concomitant use of drugs that affect CYP2D6 and/or CYP3A substrate metabolism	Reduction of glycosphingolipids. Improvements in platelet and hemoglobin levels, spleen and liver volumes and bone outcomes. No therapeutic effect in CYP2D6 ultra-rapid metabolizers
Fabry disease	Migalastat	Chaperone	Nasopharyngitis and headache were frequently reported	Decreased left ventricular mass index; reduction in the incidence of renal, cardiac or cerebrovascular events. Efficacy is restricted to patients with amenable mutations
Niemann- Pick type C	Miglustat	Substrate reduction therapy	Osmotic diarrhea and weight loss observed in the majority of the patients. Peripheral neuropathy and tremor may occur	Improvement in horizontal saccadic eye movement velocity and stabilization of ambulation, manipulation, language and swallowing scores
Cystinosis	Cysteamine	Substrate reduction therapy	Angioendotheliomatosis, unpleasant sulfurous body and breath odor, allergic rash, hyperthermia, lethargy, neutropenia, seizures and gastrointestinal discomfort were reported	Decreases extrarenal complications, delays end-stage renal disease onset, improves survival

In many LDs, symptoms are caused by the accumulation of a substrate, rather than the lack of an enzymatic product, thus having the potential to be treated by down-regulating the biosynthesis of the substrate (Coutinho *et al.*, 2016). In GD, two distinct compounds (miglustat and eliglustat), which function as glucosylceramide synthase inhibitors have been shown to have beneficial effects. Miglustat is approved for the treatment of patients with type I GD who are unable to receive ERT. Eliglustat is licensed as a first-line treatment for adult patients with type I GD (Balwani *et al.*, 2016; Belmatoug *et al.*, 2017). There is currently no consensus on whether they have the same efficacy as ERT (Zimran *et al.*, 2018). Nevertheless, the successful application of substrate reduction therapy (SRT) in the treatment of GD has encouraged the development of new small molecules or RNA-degrading technologies to achieve substrate reduction in GD and other diseases, including PD, KD, and MPS (Cabrera-Salazar *et al.*, 2012; Coutinho *et al.*, 2016; Sands and LeVine, 2016; Derrick-Roberts *et al.*, 2017; Kishnani *et al.*, 2017).

In non-enzymatic LDs, small molecules may be the only available treatment. In Niemann-Pick type C disease, in which the primary defect is in the intracellular cholesterol trafficking proteins NPC1 and NPC2, miglustat has been shown to reduce glycolipid storage in the neurons of patients. Thus, leading to improvement in horizontal saccadic eye movements, velocity and stabilization of ambulation, manipulation, language and swallowing scores (Lyseng-Williamson, 2014; Bowman *et al.*, 2017). Cysteamine, used in the therapy of nephropathic cystinosis, is the only specific treatment of this condition and is one of the first-approved LD treatments (Ariceta *et al.*, 2017). Cysteamine breaks cysteine into cysteine and cysteine-cysteamine disulfide, and it has been shown to delay the progression of renal and extrarenal disease with impacts on survival rates (Ariceta *et al.*, 2017).

Migalastat is a pharmacological chaperone (PC), which acts in patients with amenable mutations by stabilizing the enzyme alpha-galactosidase and facilitating lysosomal trafficking. It has been shown to decrease the left ventricular mass index and reduce the incidence of renal, cardiac or cerebrovascular events (Hughes *et al.*, 2017). PCs are currently being investigated in other diseases including GD and aspartylglucosaminuria (Narita *et al.*, 2016; Banning *et al.*, 2016).

Another promising class of small molecules for the treatment of LDs is the “stop codon read-through” drugs, which act by allowing the read-through of premature termination codons in patients with nonsense mutations. Currently, there are no approved drugs of this class, but ongoing clinical trials are evaluating their potential in MPS I, a condition where nonsense mutations are relatively common. Moreover, the potential of these drugs is being evaluated for other LDs, including aspartylglucosaminuria, MPS III, MPS VI, and Niemann-Pick type B (Gómez-Grau *et al.*, 2015; Banning *et al.*, 2016).

### Gene therapy and gene editing

The rationale for gene therapy for LDs is similar to the rationale for ERT, namely the ability of a deficient cell to take up a lysosomal enzyme from the extracellular milieu. This means that not all cells need to be corrected, as long as the distribution of the enzyme is efficient. Therefore, LDs are considered good targets for gene therapy, despite their multisystem involvement (Gonzalez and Baldo, 2017).

In LDs, the therapeutic goal is to achieve long-term gene expression and protein production. Therefore, most studies use vectors allowing long-term expression of the transgene, focusing particularly on lentiviruses, adeno-associated viruses, or other non-viral integrative approaches, such as gene editing (Sharma *et al.*, 2015; Beck, 2018; Schuh *et al.*, 2018).

Adeno-associated viral vectors (AAV) are emerging as the vector of choice for *in vivo* gene therapy approaches, especially for diseases with neurological involvement (Giugliani *et al.*, 2018). These viruses can transduce cells that are not going through division, and persist primarily as non-integrative episomal units, although integration has been reported (Chanda *et al.*, 2017). Pre-clinical studies in animal models have been described for most LDs, and more recently the results from the first clinical trials using these vectors were reported. Four MPS IIIA patients were treated with intracerebral injections of 7.2 10^11^viral genomes/patient of an AAV rh.10. The vector was safe and well tolerated, with one early-treated patient showing moderate improvement in neurological parameters (Tardieu *et al.*, 2014).

The use of lentiviruses in most clinical studies for LDs has focused on correcting hematopoietic stem cells (HSC) and transplanting these cells to the patient (*ex vivo* approach). A notable accomplishment, this approach was tested by injecting modified cells carrying the Arylsulfatase A (*ARSA*) gene to prevent disease manifestations in nine patients with infantile metachromatic leukodystrophy. *ARSA* expression levels were restored, and eight patients (89%) had no disease symptoms (Sessa *et al.*, 2016).

Based on the promising results from these initial trials, new studies and approaches are currently being tested. These studies include new technologies in preclinical and clinical stages, such as genome editing (Sharma *et al.*, 2015; Poswar *et al.*, 2017; Schuh *et al.*, 2018) or the use of either lentiviruses or AAV in different types of MPS and possibly other LDs (Poswar *et al.*, 2017).

## Conclusions and perspectives

Although they account for less than 1% of hereditary diseases, LDs have gained significance exceeding this proportion by concentrating a large number of successful examples of treatments for genetic conditions. Hematopoietic stem cell transplantation, enzyme replacement therapy, substrate reduction therapy, and pharmacological chaperones, are some of the approved treatment modalities that are benefiting thousands of LD patients around the world. The treatment of CNS manifestations is still a major challenge, but the administration of ERT to the brain via Trojan horses or IT/ICV, as well as gene therapy/gene editing strategies, should change this picture in the near future. Patients are usually identified after clinical suspicion, in most cases, through the identification of specific enzyme deficiencies associated with the majority of these conditions. There are a growing number of sensitive and specific biomarkers being reported that could help to screen for these conditions, support the diagnosis, and provide useful information for treatment monitoring. The development of high-throughput methods, especially based on the use of DBS, is making newborn screening feasible for several LDs. The combination of early diagnosis with effective therapies is bringing practical alternatives and hope for patients and families affected by LDs.
